# The role of *Mycobacterium tuberculosis* antigen specific cytokines in determination of acid fast bacilli culture status in pulmonary tuberculosis patients co-infected with human immunodeficiency virus

**DOI:** 10.11604/pamj.2018.31.166.17294

**Published:** 2018-11-08

**Authors:** Job Kisuya, Alex Chemtai, Evans Raballah, Wilson Okumu, Alfred Keter, Collins Ouma

**Affiliations:** 1 Department of Biomedical Science and Technology, Maseno University, Maseno, Kenya; 2 Academic Model for Providing Access to Healthcare (AMPATH), Eldoret, Kenya; 3 Department of Immunology, Moi University, Eldoret, Kenya; 4 Department of Medical Laboratory Sciences, Masinde Muliro University of Science and Technology, Kakamega, Kenya; 5 Department of Medical Biochemistry, Maseno University, Maseno, Kenya; 6 Centre for Global Health Research/Kenya Medical Research Institute, Kisumu, Kenya

**Keywords:** HIV-TB co-infection, *Mycobacterium tuberculosis*, culture, cytokines

## Abstract

**Introduction:**

The interaction between *Mycobacterium tuberculosis* and HIV leads to rapid progression of tuberculosis (TB) and human immunodeficiency virus (HIV)-induced immunosuppression. Diagnosis of TB in these patients is more difficult due to its atypical presentations giving contradicting results. The overall aim of this study was to evaluate the ability of pro-inflammatory cytokine (Th1) and anti-inflammatory cytokine (Th2) to discriminate between culture-positive and -negative smear status in HIV-TB co-infected patients.

**Methods:**

In a prospective cohort, a total of 86 study participants were recruited: 46 culture-negative and 40 culture-positive. Blood and sputum samples were collected from all participants. The blood was then analyzed using FACSCalibur flow cytometer to immunophenotype the cells and ELISA performed for cytokine profiles. Sputum samples were analyzed to determine smear status using direct microscopy and Lowenstein Jensen medium. Statistical analyses were performed using R software. Independent samples t-test was used to compare means between the two groups, while the medians were compared using two-sample Wilcoxon rank sum test. Pearson's Chi-square test was used to compare the proportion of male and female participants across the culture and AFB smear status. In order to determine the predictive power of Th1 and Th2 in discriminating Pulmonary Tuberculosis status (PTB) (culture status was used as a confirmatory test), binary logistic regression models were fitted for Th1 covariates [IFN-γ, TNF-α, IL-2 and IL-12(p70)] and Receiver Operating Characteristic (ROC) curves plotted.

**Results:**

The overall mean age of the participants was 39 years (SD=12), 42% being male. Although, lymphocytes counts were higher in culture-positive relative to culture-negative, the CD8, CD19, and CD16/CD56 were comparable in the two groups. The CD4 counts differed between the two groups (P=0.012). The Th1 showed a better discrimination between culture-positive and -negative PTB individuals; IFN-γ (P=0.001), TNF-α (P=0.001), IL-2 (P=0.001) and IL-12(p70) (P=0.016). The Th2 cytokines (IL-4, IL-6 and IL-10) were comparable between the culture-positive and -negative groups. However, when the combination of Th1 cytokines [IFN-γ, TNF-α, IL-2 and IL-12(p70)] was fitted in binary logistic regression models, the predictive power was high with area under curve (AUC) being 89.7% in discriminating PTB.

**Conclusion:**

This study provides evidence for the ability of a combination of Th1 cytokines in discriminating against culture-positive and culture-negative PTB.

## Introduction

Tuberculosis (TB) is one of the most common opportunistic infections in people infected with human immunodeficiency virus or acquired immunodeficiency syndrome (HIV and AIDS). Moreover, it is the leading cause of mortality and morbidity amongst persons with HIV and AIDS [1]. In 2014, an estimated 9.6 million people were infected with TB according to WHO-2015 global TB report, with 1.5 million people dying from the disease; 1.1 million were HIV-negative and 0.4 million HIV-positive [1]. Previous studies have revealed that one in every three person who died of AIDS was due to TB infection [2]. As such, delayed diagnosis of TB and initiation of treatment after more than three weeks of presentation was responsible for 45-85% of deaths reported among HIV-infected patients [3]. The clinical presentation of TB is often unmodified in HIV-infected immune-competent individuals. As HIV infection progresses, the immune responses weaken resulting in attenuation of host tissue-damaging responses and subsequent failure of *Mycobacterial* containment. This increases the likelihood of atypical presentation in these patients with greater proportions of extra-pulmonary and disseminated disease being reported [4]. This necessitates the need for further sputum samples examination even if the chest radiograph was normal. This observation is due to the fact that pulmonary cavitations are less common in HIV-positive patients consequently reducing the sensitivity of sputum microscopy for acid-fast bacilli (AFB) significantly [3]. Furthermore, it has been demonstrated that diagnosis by culture is time-consuming [5]. It is thus critical that emphasis is put on early detection and accurate diagnosis of *M. tuberculosis* among HIV-infected patients. This would go a long way in instituting a timely clinical management and control of the disease and eventually improvement on the survival rates among patients with PTB and HIV co-infected.

Cellular and humoral immune responses play a crucial role in controlling *M. tuberculosis* replication. The CD4 T cells through interferon gamma (IFN-γ) responses facilitate the cytotoxic functions of CD8 T cells in the host to block the development of *M. tuberculosis* disease [6]. The Th1 CD4 T cells play an important role in protection against TB by promoting activation of macrophages through the production of IFN-γ, IL-2 and Tumor Necrosis Factor alpha (TNF-α) [7, 8]. Thus, it has been postulated that perturbation in Th1 activation mechanism may lead to enhanced development of TB [9]. The CD4 depletion during the course of HIV or functional neutralization of TNF-α as a result of anti-TNF-α antibodies increases the risk of an individual to develop TB [10]. In addition, the differential expression of the Th1 cytokines (such as TNF-α, IFN-γ, IL-2, IL-8 and IL-12p70) as well as Th2 cytokines (like IL-10, IL-6 and IL-4) are associated with active TB [11]. Therefore, there is need to have distinct cytokine profiles in order to diagnose active TB. Hence, the present study was designed to evaluate the relationship between Th1 [IFN-γ, TNF-α, IL-2, IL-8 and IL-12(p70)] and Th2 (IL-4, IL-6, and IL-10) cytokine responses in culture smear-positive and -negative PTB patients co-infected with HIV.

## Methods

**Study setting:** The study was carried out at Academic Model for Providing Access to Healthcare (AMPATH) based in Western Kenya. AMPATH is a collaboration among Moi University School of Medicine (MuSoM), Moi Teaching and Referral Hospital (MTRH) and a consortium of North American led by Indiana University [12]. Previously, AMPATH focused on the delivery of HIV care, but over the past several years, it has broadened its services to include primary health care and chronic disease management, including prevention, diagnosis and treatment services for TB.

**Study population:** A prospective cohort of individuals newly diagnosed with pulmonary TB and HIV attending clinics at MTRH and AMPATH. The inclusion criteria were patients aged over 18 years, without prior history of TB or any TB relevant clinical or radiological findings or relapse. Patients who had one or more TB specific symptoms and signs based on 2013 guidelines for management of TB and leprosy in Kenya which were adopted from WHO guidelines on TB were eligible for the study [13]. Recruited study participants were those who voluntarily accepted to be tested for HIV and were naïve for highly active antiretroviral therapy (HAART) and anti TB treatment. Sputum smear-positive and -negatives patients by Ziehl-Neelsen staining were cultured to determine the presence or absence of AFB. Patients who were pregnant, HIV-negative, diabetic, or otherwise immunologically challenged or harboring an autoimmune disease were excluded from the study. These mentioned medical conditions and diseases are associated with the modification of immune responses, and therefore could alter the study's findings and conclusions. The total sample size required to attain a power of 80% to detect an effect size of 0.65, where the estimated IFN-γ mean for the culture negative is 12.99 ± 5.7 pg/ml, and estimated IFN-γ mean for the culture positive is 48.69 ± 28.78 [14], is a minimum of 72 i.e. 36 culture-positive and 36 culture-negative. Following this criteria, the total sample size for our study was 86 i.e. 40 in the culture-positive group and the 46 in the culture-negative group.

### Laboratory procedures

**Diagnosis of TB:** Sputum and blood samples collected from the study participants for TB diagnosis were analyzed as follows

**Ziehl neelsen direct microscopy:** Sputum samples collected from the study participants were smeared and stained by Ziehl Neelsen (ZN), observed for the presence or absence of acid-fast bacilli under a light microscopy with a 100X objective lens under oil immersion.

**Culturing-(lowenstein-jensen medium) and staining:** All sputum samples collected from study participants were cultured in Lowenstein Jensen (LJ) Medium for 42 days to confirm the presence or absence of acid fast bacilli. This was done irrespective of their initial ZN direct microscopy smears outcome. Positive cultures were confirmed by ZN staining and species determined by capilia (FIND and Tauns Co. Ltd) according to manufacturer's instructions. We stratified the study participants into PTB culture-positive and -negative based on post-culture staining outcome. We chose culture, because of its sensitivity and being the gold standard in the diagnosis of TB.

**Interferon Gamma Release Assay (IGRA):** IGRA was performed using quantiFERON-TB Gold-in-Tube (QFT-GIT) (Cellestis, Qiagen GmBH, Carnegie, Victoria, Australia, Catalogue 05900301) kit as per the manufacturer's instructions. One mL of blood was placed into each of the three tubes that were pre-coated with either TB antigen, phytohemaglutinin for positive control or nil antigen for negative control. The tubes were then incubated for 16-24h at 37°C and the cultured supernatant (plasma) were harvested after centrifugation and snap frozen at -80°C until use.

**Measurement of cytokines:** The cytokines levels for IL-2, IL-4, IL-6, IL-8, IL-10, IL-12 (p70), TNF-α and IFN-γ in QFT-GIT supernatants were measured by standard sandwich ELISA technique using GenWays Biotech Kit (Greenways Biotech Inc., San Diego, CA, USA; Catalogue #s GWB-ZZD007, GWB-ZZD002, GWB-ZZD006, GWB-ZZD013, GWBZZD005, GWBZZD009, GWBZZD003 and GWB-ZZD004) as per the manufacturer's instructions. In brief, the ELISA kit was based on standard sandwich enzyme-linked immune-sorbent assay technology. The test cytokine specific monoclonal antibodies pre-coated to the plate and the human specific detection polyclonal antibodies were biotinylated. The QFT-GIT supernatant and biotinylated detection antibodies were added to the wells subsequently and followed by washing with 0.01M Phosphate Buffered Solution (PBS). Avidin-Biotin-Peroxidase Complex was added and unbound conjugates were washed away with 0.01M PBS. Horseradish peroxidase substrate (HRP) 3,3',5,5'-tetramethylbenzidine (TMB) was used to visualize HRP enzymatic reaction. TMB was catalyzed by HRP to produce a blue color product that changed into yellow after adding acidic stop solution. The Optical Density (OD) of each well were measured within 5 minutes of stopping the reaction using an ELISA microplate reader fitted with a 450nm filter and with a 620nm reference filter. The OD absorbance was read at 450nm. Cytokines concentrations were measured in OD.

**Immunophenotyping:** Whole blood samples were collected into 4mL EDTA-containing vacutainer tubes (Becton Dickinson, Franklin Lakes, NJ, USA). Determination of lymphocyte subsets was performed using a FACSCalibur flow cytometer (Becton Dickinson Immunocytometry Systems, San Jose, CA, USA) on single technology platform. Two combinations of 4 monoclonal antibodies were used (anti-CD3/CD8/CD45/CD4 and anti-CD3/CD16+56/CD45/CD19 Catalogue # 342416) and TruCOUNT tubes (Becton Dickinson, NJ, USA). About 20μL of MultiTEST CD3FITC/CD8PE/CD45PERCP/CD4APC (Becton Dickson, NJ, USA, Catalogue # 342417) reagent was pipetted into the bottom of one tube while another 20μL of MultiTEST CD3FITC/CD16+CD56PE/CD45PERCP/CD19APC into the bottom of a second tube. About 50 μL of well-mixed, anti-coagulated whole blood sample was pipetted into the bottom of each tube. The tubes were capped and vortex gently to mix, then incubated for 15 minutes in the dark at room temperature (20-25°C). Following this step, 450 μL of the Lysing Solution (Becton Dickson, NJ, USA, Catalogue #349202) was added into each tube. The tubes were again capped and vortex gently to mix and incubated for 15 minutes in the dark at room temperature (20-25°C). Lymphocyte subsets were enumerated using MultiSET software (Becton Dickinson, NJ, USA).

**Statistical analysis:** Data analysis was performed using software for statistical computation (R Core Team, 2016). Categorical variables such as gender were summarized as frequencies and their corresponding percentages. Continuous variables were assessed for Gaussian assumptions using Shapiro Wilks test and normal probability plots. Those that met the Gaussian assumptions were summarized as mean and the corresponding standard deviation (SD), while those that violated the assumptions were summarized as median and their corresponding interquartile range (IQR). Means between two groups were compared using independent sample t-test. The medians were compared using two-sample Wilcoxon rank sum test. Pearson's Chi-square test was used to compare the distribution of male and female participants across the culture and AFB smear status. In order to assess the predictive ability of Th1 and Th2 in the diagnosis of the PTB status (culture status used as the confirmed outcome), binary logistic regression models were fitted for Th1 (IFN-γ, TNF-α, IL-2 and IL-12(p70) and Receiver Operating Characteristic (ROC) curves plotted. The area under the curve (AUC) was calculated to assess the predictive power of the model in giving us the correct diagnosis. Statistical significance was assessed at P≤0.05.

## Results

**Demographic characteristics and clinical characteristics (lymphocyte proportion) of the PTB culture-positive and -negative study participants:** A total of 86 participants met the inclusion criteria and were enrolled into the study. These were partitioned as follows: PTB culture-positive (n=40) and PTB culture-negative (n=46). The overall mean age was 39 (SD=12) years. The mean age for males study participants was 36.6 (SD=12.0) years with almost half (45.7%) being culture-negative smears and more than half (54.3%) of the females were culture-negative. The demographic characteristics, age and gender were comparable between PTB-positive and PTB-negative (Table 1). The study further sought to determine the relationship between lymphocyte proportions in PTB culture-positive and -negative participants. The results presented here demonstrated that CD8 (P=0.979), CD19 (P=0.346), and CD16/CD56 (P=0.637) were comparable between the two groups. Although, in general the median counts of T lymphocytes were higher in culture-positive as compared to culture-negative participants, it is only median CD4 count of 504.5 (IQR: 217.5, 907.5) vs. 473.0 (IQR: 157.8, 1166.0), respectively, that differed (P=0.012) between the groups as shown in Table 1.

**Table 1:** Demographic and clinical characteristics between the PTB culture-positive and -negative of study participants

		Culture Results		
	N	Negative (N=46)	Positive (N=40)	
		Median (IQR) or n (%)	Median (IQR) or n (%)	P-value
Age	86	41.2(12.0)	36.6(11.7)	0.078^a^
Male	86	21 (45.7)	21(52.7)	0.526^b^
CD8	86	473.0 (157.8, 1166.0)	504.5 (217.5, 907.50)	0.979^c^
CD4	86	67.5 (22.5, 192.5)	210.5 (97.3, 309.5)	0.012^c^
CD16/CD56	86	63.0 (33.3, 113.5)	87.0 (46.5, 138.0)	0.346^c^
CD19	86	46.0 (15.5, 99.3)	46.0 (21.8, 129.8)	0.637^c^

**Tuberculosis diagnoses results:** The following three approaches were used to diagnose for TB; direct sputum staining by ZN and microscopy, culturing of sputum, subsequent staining by ZN and speciation by capila and lastly IGRA. In the direct staining of sputum by ZN, 16 individuals were diagnosed as positive, while 70 were diagnosed as negative for *M. tuberculosis*. When, the sputum samples were first cultured in *Lowenstein-Jensen Medium, with ZN* and speciated with capila, 40 participants tested positive while 46 were negative for *M. tuberculosis*. Finally, the IGRA showed that 37 participants were indeterminate, 21 were negative and 28 were positive for *M. tuberculosis*. Based on these results, study participants were categorized to be *M. tuberculosis* positive or negative based on the gold standard culture and subsequent staining (40 positive and 46 negative) (Table 2). In addition, we compared IFN-γ production between the *M. tuberculosis* positive and negative participants from the IGRA results. Prior to this, the cytokine values from nil tube were subtracted from the TB antigen tube concentrations. Results presented here, demonstrate that participants who were positive for *M. tuberculosis* had elevated IFN-γ levels (P<0.001) (Table 3).

**Table 2:** Tuberculosis diagnosis

Test/Results	IGRA	Sputum ZN staining	Culture and staining
Indeterminate	37	-	-
Positive	21	16	40
Negative	28	70	46

**Table 3:** Comparison of cytokines levels between TB positive and negative participants

			Culture status		
		Total	Negative	Positive	
	N	Median (IQR)	Median (IQR)	Median (IQR)	P
IFN-γ	86	4.4 (0.0, 34.0)	0.0 (-1.9, 4.0)	37.0 (7.1, 138.9)	<0.001
TNF-α	85	-0.4 (-2.9, 2.5)	-1.2 (-2.9, 1.2)	1.4 (-3.3, 3.5)	0.069
IL-2	86	7.5 (-3.8, 40.5)	3.9 (-5.3, 8.5)	42.9 (5.3, 133.0)	<0.001
IL-4	86	0.0 (-4.2, 11.5)	-1.0 (-5.1, 3.6)	5.3 (-3.3, 12.7)	0.046
IL-6	86	0.8 (-9.3, 9.1)	1.7 (-8.4, 10.6)	-1.6 (-9.7, 7.2)	0.346
IL-8	82	-26.5 (-98.2, 32.7)	-26.5(-74.8, 27.4)	-27.2 (-101.9, 51.9)	0.993
IL-10	86	0.9 (-2.0, 3.5)	1.4 (-0.3, 4.3)	0.3 (-4.9, 3.0)	0.011
IL-12	82	0.7 (-0.4, 2.0)	0.6 (-0.4, 1.9)	0.7 (0.0, 2.2)	0.375

**Relationship between Th1 cytokines and PTB culture status of study participants:** To further extend these studies, we determined the relationship between Th1 Cytokines and TB culture status (Table 4). Results revealed that the Th1 cytokines levels, IFN-γ (P=0.001), TNF-α (P=0.001), IL-2 (P=0.001) and IL-12(p70) (P=0.016) were elevated in culture-positive participants compared to culture-negative participants, however, IL-8 (P=0.337) were non-significantly higher in PTB culture-positive (Table 4).

**Table 4:** Relationship between Th1 cytokines and PTB culture status of study participants

		Culture Results		
	N	Negative (N=46)	Positive (N=40)	
		Median (IQR) or n (%)	Median (IQR) or n (%)	*P*-value*
IFN-γ	86	7.6 (5.6, 16.9)	56.0 (29.8, 167.0)	0.001
TNF -α	86	16.1 (14.4, 19.2)	21.0 (16.8, 24.6)	0.001
IL-2	86	15.3 (8.4, 26.9)	59.6 (23.9, 145.6)	0.001
IL-12(p70)	82	2.1 (0.7, 3.5)	3.1 (1.9, 5.0)	0.016
IL-8	82	154.2 (50.1, 204.5)	126.6 (72.5, 167.6)	0.337

**Relationship between Th2 Cytokines and PTB culture status of study participants:** In order to explore the relationship between Th2 cytokines and PTB, we performed two-sample Wilcoxon rank sum test. Results presented here show that the Th2 cytokines median levels between the culture-positive and culture-negative participants were comparable, IL-4 median level of 8.6 (IQR1.1, 26.0) vs. 18.9 (IQR 3.4, 52.7), IL-6 median level of 10.1 (IQR 34.0, 44.0) vs. 12.1 (3.9, 27.6), and IL-10 median level of 10.8 (IQR 8.6, 13.7) vs. 12.8 (IQR 8.4, 15.7) (Table 5).

**Table 5:** Relationship between Th2 cytokines and PTB culture status participants

		Culture Results		
	N	Negative (N=46)	Positive (N=40)	
		Median (IQR) or n (%)	Median (IQR) or n (%)	P-value
IL-4	86	8.6 (1.1, 26.0)	18.9 (3.4, 52.7)	0.063
IL-6	86	12.1 (3.9, 27.6)	10.1 (34.0, 44.0)	0.891
IL-10	86	12.4 (8.4, 15.7)	10.8 (8.6, 13.7)	0.187

**Figure 1:**
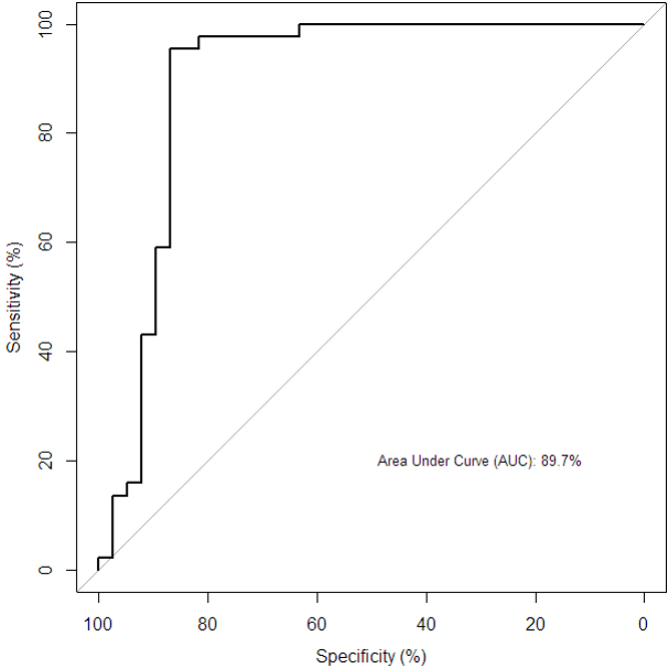
Predictive power of Th1 on culture status among all the participants

**Figure 2:**
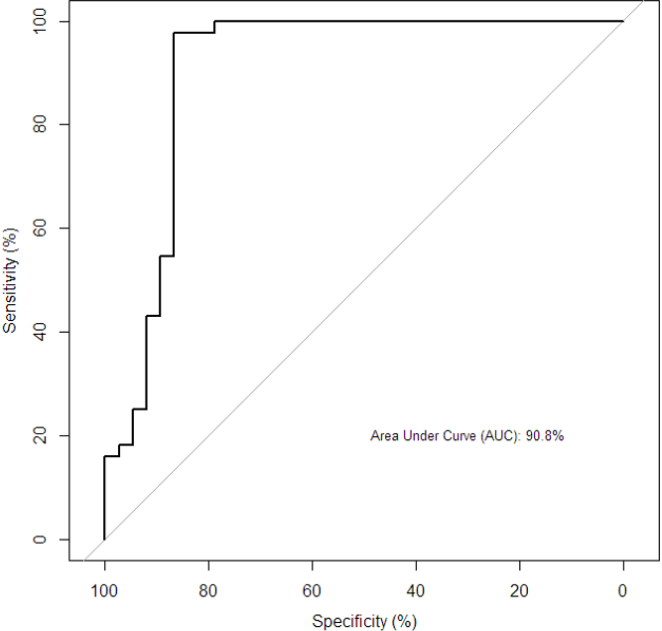
Predictive power of Th1 in combination with AFB smears status on culture status among all the participants

**Predictive power of Th1 cytokine on culture:** To elucidate the predictive power of Th1 cytokine on culture status, we performed a ROC analysis. The results presented here reveal that the ROC, area under the curve (AUC) was 89.7% (Figure 1) which indicated an excellent predictive power of the model with Th1 [IFN-γ, TNF-α, IL-2 and IL-12(p70)] in discriminating between culture-positive and culture-negative in PTB patients. When culture AFB smear status was combined with the selected Th1 cytokines, the AUC was 90.8% as shown in Figure 2.

**Predictive power of Th2 cytokine on culture:** In order to determine the predictive power of Th2 cytokines, we performed two-sample Wilcoxon rank sum test. Our results indicate that the Th2 cytokines (IL-4, P=0.063; IL-6 P=0.891 and IL-10, P=0.187) were comparable between PTB culture-negative and -positive individuals, hence they did not have a diagnostic value to predict a TB case.

## Discussion

This current study sought to evaluate the role of *M. tuberculosis* antigens-specific cytokines in determination of smear status in pulmonary tuberculosis co-infected with HIV. This approach was in an attempt to develop a diagnostic technique that utilizes a combination of these cytokines in rapidly and accurately detecting the presence or absence of TB. The most commonly available methods used in the detection of the bacilli have been shown to be less sensitive, less accurate or takes a longer period to carry out a diagnosis [1, 5]. Similar to other studies carried out, we observed a CD4 T-lymphocytopenia in TB-HIV co-infected patients as compared to HIV-infected individuals [15]. The CD4 T lymphocytes are known to confer protection against *M. tuberculosis* through induction of IFN-γ and TNF-α by recruiting of monocytes and granulocytes and their anti-microbial activities [16, 17]. Thus, this observation does not show a direct correlation of HIV-TB co-infection disease progression with CD4 levels in an individual, which further support argument that HIV disease progression monitoring on the account of CD4 counts, might be misleading in HIV-TB co-infection.

In the current study, the levels of IFN-γ were significantly high in PTB culture-positive as compared to PTB culture-negative study participants. These findings corroborate the findings of a previous study in which it was indicated that patients with culture-confirmed PTB positives had higher levels of IFN-γ [18]. This was irrespective of HIV status of an individual. These observations indicate the potential of IFN-γ to discriminate between PTB culture-positive and PTB culture-negative after stimulation with MTB-specific antigens. We further assessed the association between TNF-α and PTB culture status. TNF-α is a Th1 cytokine known to play a key role in the formation of granuloma for the containment and protection against *Mycobacterium*. Reports from previous studies have shown elevated levels of TNF-α in culture supernatants from stimulated whole blood with TB-specific antigens (ESAT-6, CFP-10 and TB7.7 present in QFT-IT assay), and this was in agreement with the current study [19]. In the current study, there were variations in TNF-α levels between PTB culture-positive and PTB culture-negative, with higher levels of the cytokine being observed in PTB culture-positive. These results suggest that TNF-α levels are elevated during TB infection. It is plausible that the enhanced production contributes to the formation of granuloma, an important immunological event for the containment and protection against *Mycobacterium*. Consequently, other studies in western Africa and India have demonstrated that the TNF-α levels can be used to distinguish between individuals with and without TB infection [20, 21]. IL-2 plays a critical role in the activation, proliferation and clonal expansion of T lymphocyte. The results from the current study indicated that IL-2 levels were higher in culture-positive in relation to the culture-negative individuals. Previous investigations support our current observations [20]. It has been shown, that IL-2 reduces the replication of *Mycobacterium* through activation of CD8 cytotoxic T lymphocyte and the activation of macrophages to release interferons [22]. In this regard, we hypothesize that higher levels of IL-2 reported in culture-positive individuals could be due to presence of the MTB antigens, hence, the differing levels of this cytokine between the two groups.

Moreover, a study carried in India, reported lower levels of IL-12p70 in HIV patients when compared to TB-HIV co-infected individuals [23], an observation that was consistent with findings of the present study. Although the difference in IL-12(p70) cytokines levels between groups was small in the present study, they were still statistically significant. It is important to note that IFN-γ and IL-12(p70) mutually influence their own production, therefore, indicating the ability of IL-12(p70) in discriminating between culture-positive and -negative individuals. It is also important to note that combination of the Th1 cytokines [IFN-γ, TNF-α, IL-2 and IL-12(p70)] showed an excellent predictive power in discriminating between cultures-confirmed pulmonary-tuberculosis patients against culture-negative individuals. Similar results were reported by a study carried out in China, although the AUC was slightly higher compared to the current study [24]. This could be attributed to the difference in the methods used, whereas in the previous study pleural effusion mononuclear cells were stimulated with *Mycobacterium tuberculosis* specific antigen while in the present study, whole blood was stimulated with TB-specific antigens. Despite the differences in the methodology, it was still evident that Th1 cytokines were predominant in discriminating between the two groups.

## Conclusion

In conclusion, our study provides evidence that the combination of Th1 cytokines [IFN-γ, TNF-α, IL-2 and IL-12(p70)] have a potential to discriminate between culture-positive and -negative pulmonary tuberculosis with HIV co-infection. Incorporating a combination of this group of cytokines has the potential of improving early and accurate diagnosis of *Mycobacterium tuberculosis*.

### What is known about this topic

- Studies have shown ability of interferon-gamma to diagnosis pulmonary tuberculosis in immune-competent individuals.

### What this study adds

- This study provides evidence for the ability of a combination of cytokines in discriminating against culture-positive and culture-negative PTB in HIV co-infected individuals.

## Competing interests

The authors declare no competing interests.
